# Supplementary material for: Rapid evolutionary diversification of the flamenco locus across simulans clade Drosophila species
Source: PLoS Genet. 2023 Aug 29;19(8):e1010914. doi: 10.1371/journal.pgen.1010914 (PMC10495008; doi:10.1371/journal.pgen.1010914)
Supplement: S1 File — Because piRNA clusters are so difficulty to assemble, we use an approach here called Cluster Busco. Essentially the rate of soft clipping and coverage are calculated for BUSCO genes. These are then compared to the piRNA clsuters to look for regions with considerably different coverage/soft clipping than BUSCO genes. Here are 99% quantiles for BUSCO genes indicated by the dotted lines for both coverage and soft clipping. Then the rate of soft clipping and coverage are shown as the black line for each assembly. In some cases the assembly was modified based off this information–for example NS40 has a spike in coverage/soft clipping which was an assembly error. The flamenco region in NS40 actually ends at that position. (PDF) [file pgen.1010914.s001.pdf]

Supplementary File 1: Coverage and soft clipping are both good indicators of assembly quality. Because piRNA clusters are so difficult to assemble, we use an approach here called Cluster Busco. Essentially the rate of soft clipping and coverage are calculated for BUSCO genes. These are then compared to the piRNA clusters to look for regions with considerably different coverage/soft clipping than BUSCO genes. Here are 99% quantiles for BUSCO genes are indicated by the dotted lines for both coverage and soft clipping. Then the rate of soft clipping and coverage are shown as the black line for each assembly. In some cases the assembly was modified based off of this information - for example NS40 has a spike in coverage/soft clipping which was an assembly error. The flamenco region in NS40 actually ends at that position.

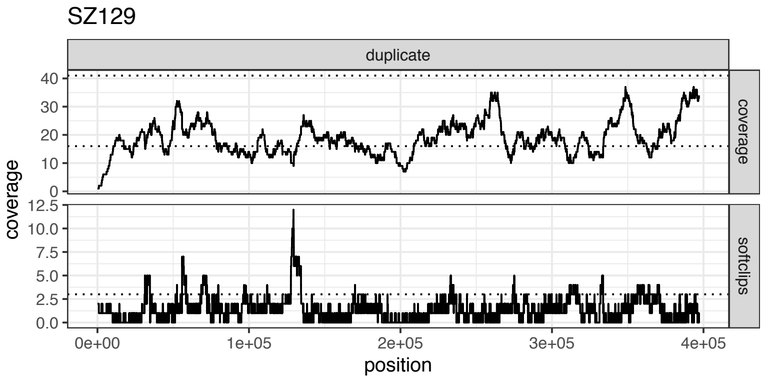

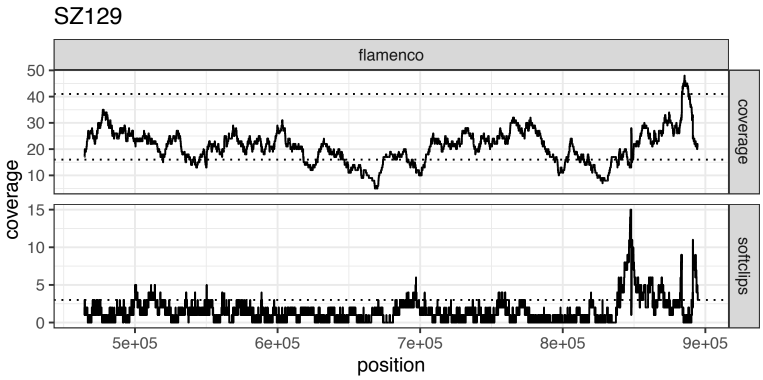

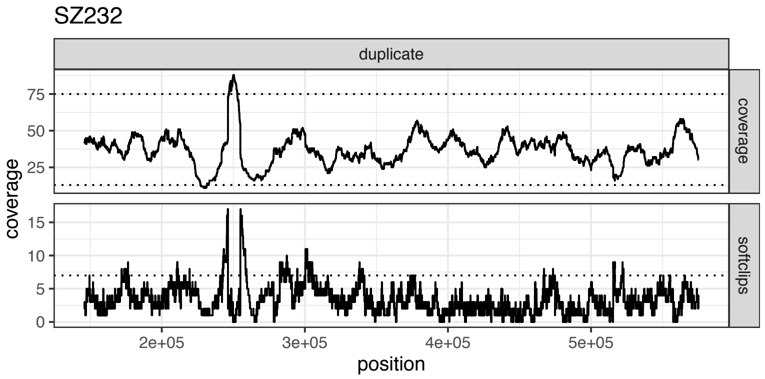

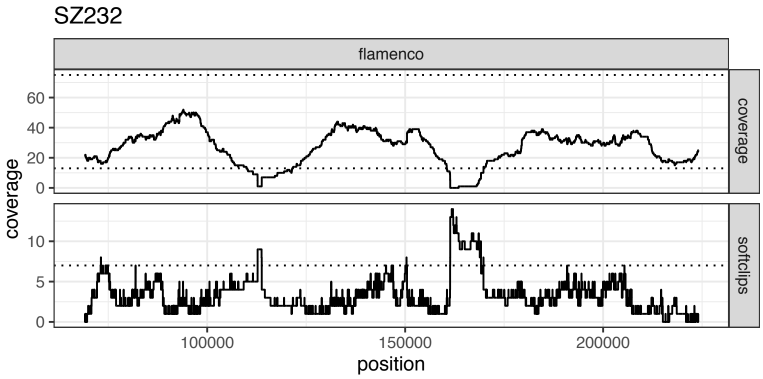

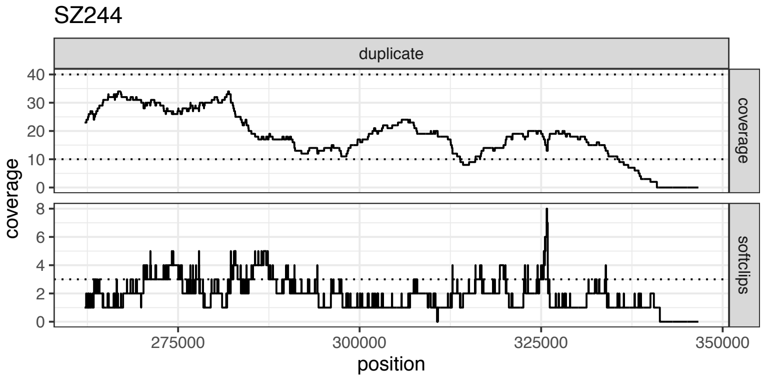

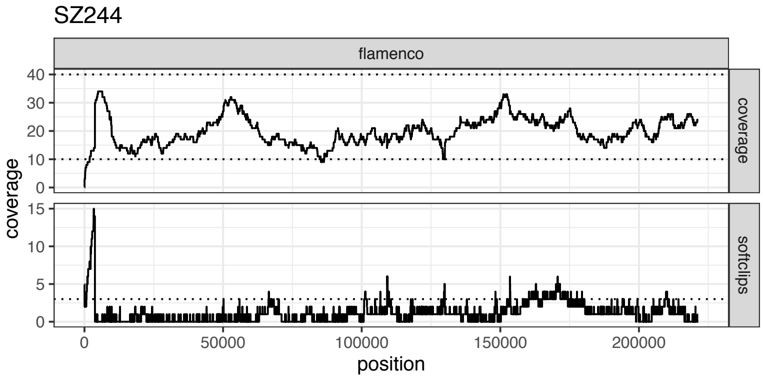

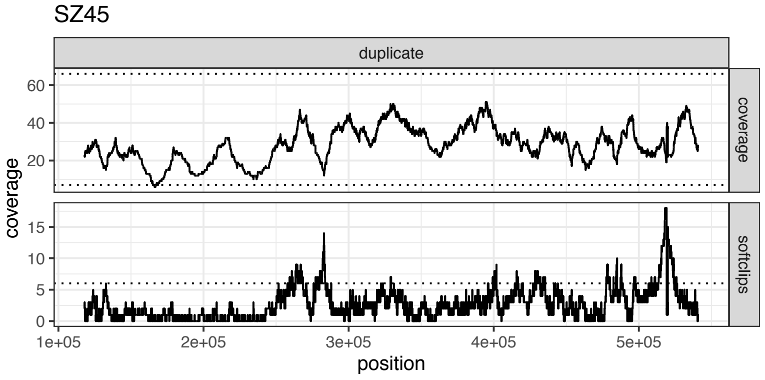

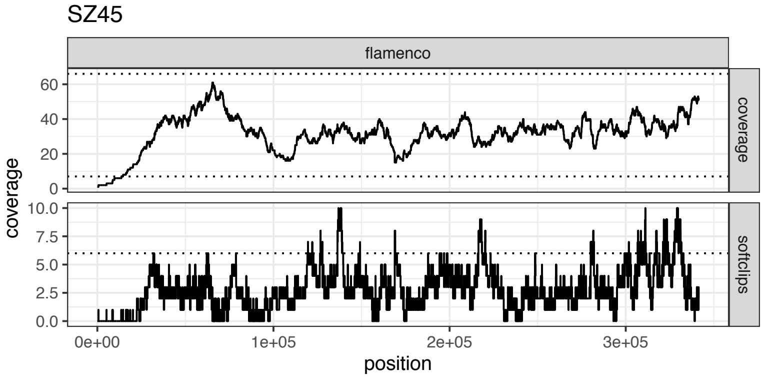

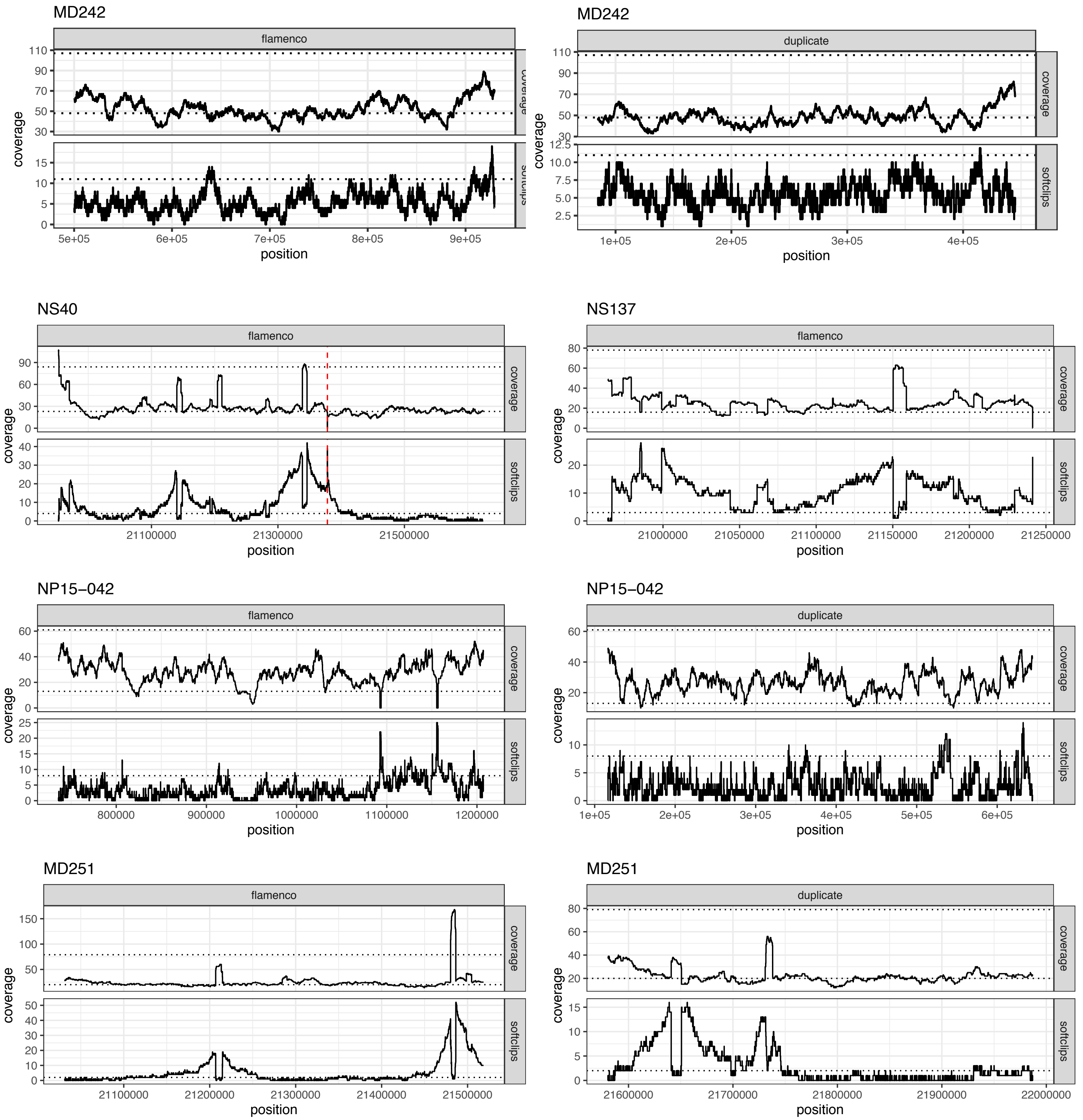

MD242
flamenco
coverage
softclips
position
MD242
duplicate
coverage
softclips
position
NS40
flamenco
coverage
softclips
position
NS137
flamenco
coverage
softclips
position
NP15–042
flamenco
coverage
softclips
position
NP15–042
duplicate
coverage
softclips
position
MD251
flamenco
coverage
softclips
position
MD251
duplicate
coverage
softclips
position

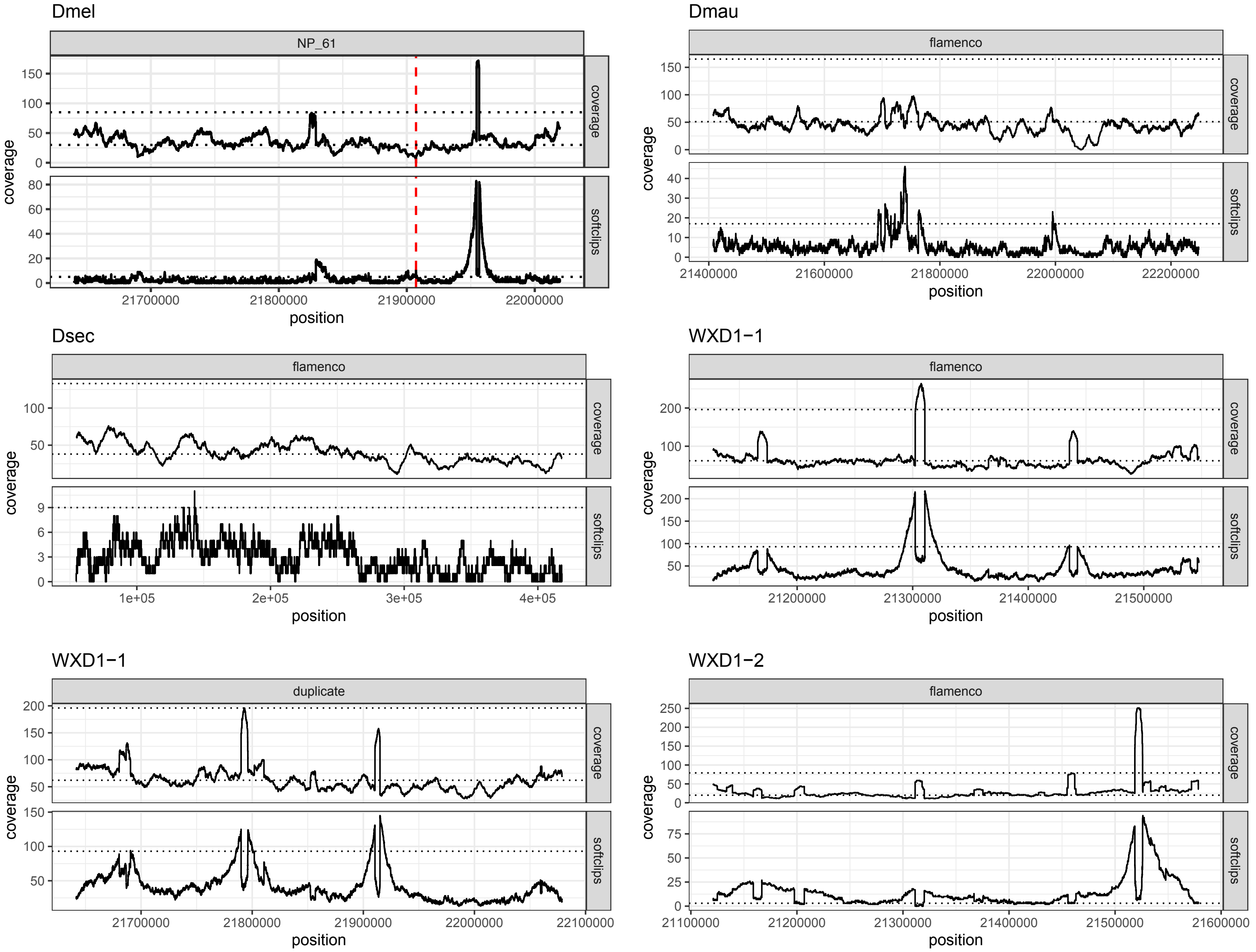
Dmel
NP_61
coverage
softclips
position
Dmau
flamenco
Dsec
WXD1−1
duplicate
WXD1−2
