# Supplementary material for: Rapid evolutionary diversification of the flamenco locus across simulans clade Drosophila species
Source: PLoS Genet. 2023 Aug 29;19(8):e1010914. doi: 10.1371/journal.pgen.1010914 (PMC10495008; doi:10.1371/journal.pgen.1010914)
Supplement: S2 File — (PDF) [file pgen.1010914.s002.pdf]

Duplicate confirmation primers

flamenco_F	CAACCAGAGATGGCTAGCACTG
flamenco_R	GCTGGCACTGGTACTGGC

Restriction enzyme: BsaAI
