# Supplementary material for: Rapid evolutionary diversification of the flamenco locus across simulans clade Drosophila species
Source: PLoS Genet. 2023 Aug 29;19(8):e1010914. doi: 10.1371/journal.pgen.1010914 (PMC10495008; doi:10.1371/journal.pgen.1010914)
Supplement: S3 File — This example is from LNP-15-062. The chromosome, start, and end of the 28S rDNA is listed first, followed by the chromosome, start, and end of the R1 insertion. The last column corresponds to the length of the R1 TE. A full length R1 element is about 5429 bp long. (PDF) [file pgen.1010914.s003.pdf]

Chr	Start	End	rDNA	Chr	Start	End	TE	Length
tig00000238	140612	141867	28S_rDNA	tig00000238	141868	147297	R1_DSi	5429
tig00000238	147298	149998	28S_rDNA	tig00000238	141868	147297	R1_DSi	5429
tig00000722	15965	17217	28S_rDNA	tig00000722	17218	22638	R1_DSi	5420
tig00000722	22639	25337	28S_rDNA	tig00000722	17218	22638	R1_DSi	5420
tig00000726	39225	40478	28S_rDNA	tig00000726	40479	45897	R1_DSi	5418
tig00000726	45898	48594	28S_rDNA	tig00000726	40479	45897	R1_DSi	5418
tig00000256	16894	18160	28S_rDNA	tig00000256	11486	16903	R1_DSi	5417
tig00000256	11431	11485	28S_rDNA	tig00000256	11486	16903	R1_DSi	5417
tig00000754	53972	55229	28S_rDNA	tig00000754	55230	60644	R1_DSi	5414
tig00000724	56934	59629	28S_rDNA	tig00000724	51519	56933	R1_DSi	5414
tig00000754	60645	63342	28S_rDNA	tig00000754	55230	60644	R1_DSi	5414
tig00000724	50447	51506	28S_rDNA	tig00000724	51519	56933	R1_DSi	5414
tig00000724	66739	67995	28S_rDNA	tig00000724	67996	73409	R1_DSi	5413
tig00000724	73410	76099	28S_rDNA	tig00000724	67996	73409	R1_DSi	5413
tig00000725	3783	5035	28S_rDNA	tig00000725	5036	10448	R1_DSi	5412
tig00000725	10449	13147	28S_rDNA	tig00000725	5036	10448	R1_DSi	5412
tig00000668	59684	60793	28S_rDNA	tig00000668	55057	59693	R1_DSi	4636
tig00000227	44947	46054	28S_rDNA	tig00000227	40329	44956	R1_DSi	4627
tig00000244	50502	51761	28S_rDNA	tig00000244	51762	55785	R1_DSi	4023
tig00000754	70166	71416	28S_rDNA	tig00000754	71417	74948	R1_DSi	3531
tig00000244	23841	25099	28S_rDNA	tig00000244	25100	28035	R1_DSi	2935
tig00000256	52598	52715	28S_rDNA	tig00000256	50099	52599	R1_DSi	2500
tig00000244	37156	37207	28S_rDNA	tig00000244	34675	37155	R1_DSi	2480
tig00000244	2297	4993	28S_rDNA	tig00000244	1	2296	R1_DSi	2295
tig00000669	52569	52622	28S_rDNA	tig00000669	52623	54864	R1_DSi	2241
tig00000669	32481	32534	28S_rDNA	tig00000669	32535	34775	R1_DSi	2240
tig00000669	23862	23916	28S_rDNA	tig00000669	23917	26156	R1_DSi	2239
tig00000750	9093	9147	28S_rDNA	tig00000750	9148	11387	R1_DSi	2239
tig00000669	80720	80774	28S_rDNA	tig00000669	80775	83013	R1_DSi	2238
tig00000751	12895	12949	28S_rDNA	tig00000751	12950	15187	R1_DSi	2237
tig00000750	18634	18687	28S_rDNA	tig00000750	18688	20920	R1_DSi	2232
tig00000752	10158	10207	28S_rDNA	tig00000752	10208	12439	R1_DSi	2231
tig00000669	56977	57031	28S_rDNA	tig00000669	57032	59257	R1_DSi	2225
tig00000750	23097	23146	28S_rDNA	tig00000750	23147	25362	R1_DSi	2215
tig00000669	20616	20647	28S_rDNA	tig00000669	18184	20378	R1_DSi	2194
tig00000669	18129	18183	28S_rDNA	tig00000669	18184	20378	R1_DSi	2194
tig00000669	20356	20547	28S_rDNA	tig00000669	18184	20378	R1_DSi	2194
tig00000669	15207	15249	28S_rDNA	tig00000669	12993	15183	R1_DSi	2190
tig00000750	4729	4783	28S_rDNA	tig00000750	4784	6974	R1_DSi	2190
tig00000669	12938	12992	28S_rDNA	tig00000669	12993	15183	R1_DSi	2190
tig00000751	3400	3439	28S_rDNA	tig00000751	3440	5606	R1_DSi	2166
tig00000227	67263	67317	28S_rDNA	tig00000227	67318	69483	R1_DSi	2165
tig00000227	69481	69988	28S_rDNA	tig00000227	67318	69483	R1_DSi	2165
tig00000669	70102	70569	28S_rDNA	tig00000669	67940	70104	R1_DSi	2164

tig00000669	67885	67939	28S_rDNA	tig00000669	67940	70104	R1_DSi	2164
tig00000751	17274	17328	28S_rDNA	tig00000751	17329	19481	R1_DSi	2152
tig00000669	89370	89424	28S_rDNA	tig00000669	89425	91571	R1_DSi	2146
tig00000256	45266	47963	28S_rDNA	tig00000256	47964	50098	R1_DSi	2134
tig00000669	48592	48638	28S_rDNA	tig00000669	48639	50745	R1_DSi	2106
tig00000751	28973	29025	28S_rDNA	tig00000751	29026	30991	R1_DSi	1965
tig00000754	25257	26510	28S_rDNA	tig00000754	26511	28298	R1_DSi	1787
tig00000754	28298	30633	28S_rDNA	tig00000754	26511	28298	R1_DSi	1787
tig00000750	14307	14361	28S_rDNA	tig00000750	14980	16736	R1_DSi	1756
tig00000669	63905	64276	28S_rDNA	tig00000669	61293	62921	R1_DSi	1628
tig00000669	62921	63425	28S_rDNA	tig00000669	61293	62921	R1_DSi	1628
tig00000669	85149	85203	28S_rDNA	tig00000669	85587	87214	R1_DSi	1627
tig00000752	2072	2126	28S_rDNA	tig00000752	2127	3651	R1_DSi	1524
tig00000751	25509	25824	28S_rDNA	tig00000751	24085	25494	R1_DSi	1409
tig00000244	61891	64586	28S_rDNA	tig00000244	60498	61890	R1_DSi	1392
tig00000726	923	3600	28S_rDNA	tig00000726	1	922	R1_DSi	921
tig00000669	76810	77312	28S_rDNA	tig00000669	75916	76812	R1_DSi	896
tig00000669	76810	77312	28S_rDNA	tig00000669	77864	78567	R1_DSi	703
tig00000750	30214	30268	28S_rDNA	tig00000750	31213	31876	R1_DSi	663
tig00000750	14307	14361	28S_rDNA	tig00000750	14362	14992	R1_DSi	630
tig00000750	30214	30268	28S_rDNA	tig00000750	30707	31212	R1_DSi	505
tig00000750	30214	30268	28S_rDNA	tig00000750	30269	30695	R1_DSi	426
tig00000669	85149	85203	28S_rDNA	tig00000669	85204	85609	R1_DSi	405
tig00000788	391	1652	28S_rDNA	tig00000788	1	400	R1_DSi	399
tig00000750	30214	30268	28S_rDNA	tig00000750	30039	30188	R1_DSi	149
tig00000249	97	150	28S_rDNA	tig00000249	1	96	R1_DSi	95

Supplemental File 3: The position of R1 insertions and 28S rDNA. This example is from LNP-15-062. The chromosome, start, and end of the 28S rDNA is listed first, followed by the chromosome, start, and end of the R1 insertion. The last column corresponds to the length of the R1 TE. A full length R1 elements is 5429 bp long.
