# Supplementary material for: Rapid evolutionary diversification of the flamenco locus across simulans clade Drosophila species
Source: PLoS Genet. 2023 Aug 29;19(8):e1010914. doi: 10.1371/journal.pgen.1010914 (PMC10495008; doi:10.1371/journal.pgen.1010914)
Supplement: S1 Fig — Unlike in D. simulans there is a stronger enrichment of ping pong scores in the testis as flamenco, though both show ping pong signals in the maternal fraction. (PDF) [file pgen.1010914.s005.pdf]

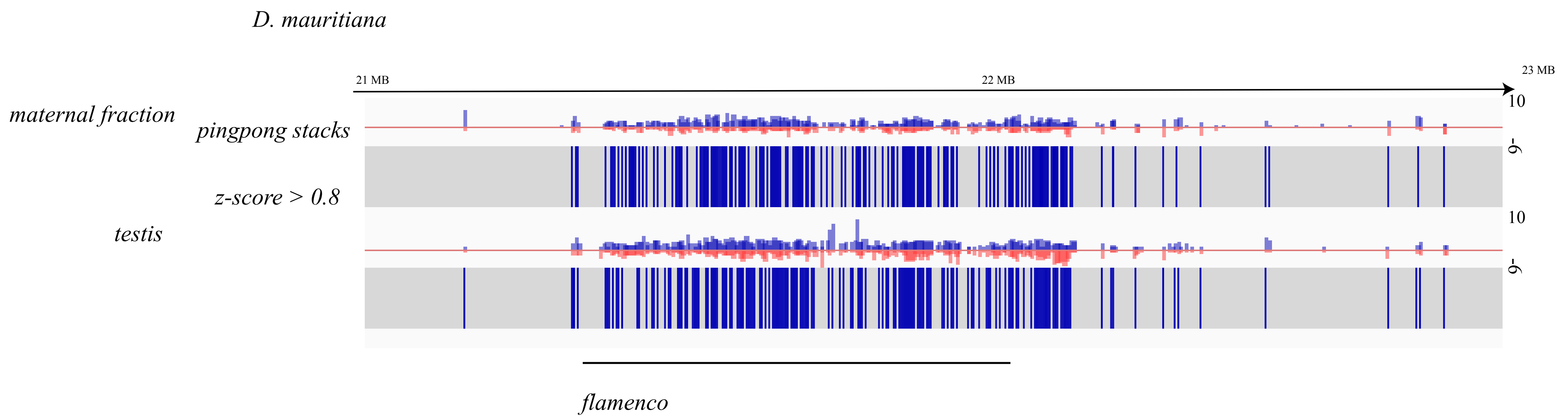

Supplementary Figure 1: The height of the ping pong stacks and the distribution of z-scores greater than 0.8 in *D. mauritiana* maternal fraction and testis. Unlike in *D. simulans* there is a stronger enrichment of ping pong scores in the testis at *flamenco*, though both show ping pong signals in the maternal fraction.
