# Supplementary material for: Rapid evolutionary diversification of the flamenco locus across simulans clade Drosophila species
Source: PLoS Genet. 2023 Aug 29;19(8):e1010914. doi: 10.1371/journal.pgen.1010914 (PMC10495008; doi:10.1371/journal.pgen.1010914)
Supplement: S2 Fig — The original copy of flamenco is on the left, the duplicate on the right. Copy number of the fragmented dip1 gene is variable between strains, LNP-15-062 having more copies than most other genotypes. Dip1 is indicated by the blue boxes, and piRNA is shown as RPM. Note that not all transposons present in flamenco are labeled, given their fragmented nature they are often very dense which would require the labels to be very crowded. Included is enough to give a general map of the area. (PDF) [file pgen.1010914.s006.pdf]

LNP-15-062

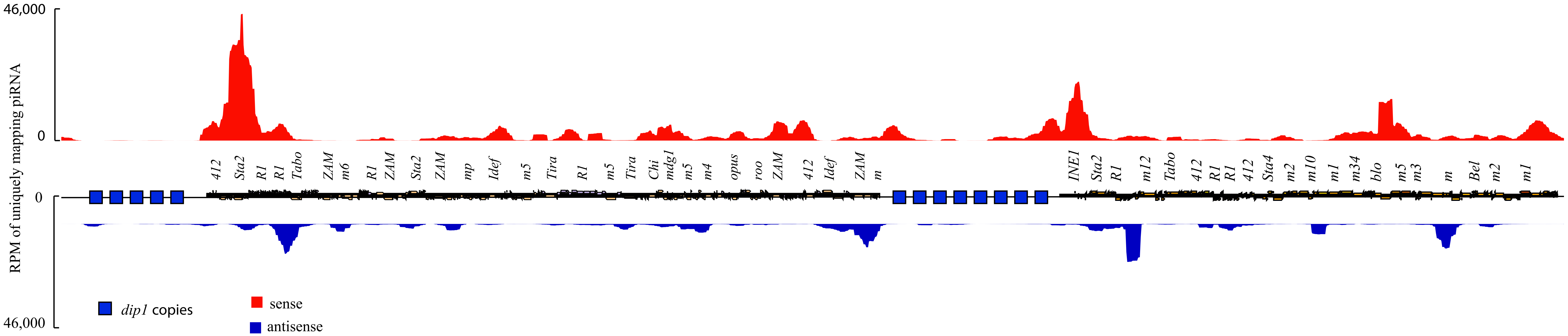

Supplementary Figure 2: A higher resolution map of the transposons and uniquely mapping piRNA for one genotype of *D. simulans*. The original copy of flamenco is on the left, the duplicate on the right. Copy number of the fragmented *dip1* gene is variable between strains, *LNP-15-062* having more copies than most other genotypes. *dip1* is indicated by the blue boxes. piRNA is shown as RPM. Note that not all transposons present in *flamenco* are labeled, given their fragmented nature this would require a lot of crowding of the labels, but enough to give a general map of the area are included.
