# Supplementary material for: Rapid evolutionary diversification of the flamenco locus across simulans clade Drosophila species
Source: PLoS Genet. 2023 Aug 29;19(8):e1010914. doi: 10.1371/journal.pgen.1010914 (PMC10495008; doi:10.1371/journal.pgen.1010914)
Supplement: S1 Table — (PDF) [file pgen.1010914.s008.pdf]

Species/Genotype	# of contigs	N50	N90	Assembly Size	Largest Contig
SZ 129	345	21.1 MB	1.1 MB	136 MB	27.6 MB
SZ 232	870	3.5 MB	51 KB	163 MB	20.3 MB
SZ 244	544	18.9 MB	100 KB	150 MB	21.2 MB
SZ 45	1281	2.7 MB	39 KB	174 MB	17.4 MB
LNP-15-062	728	4.8 MB	60 KB	151 MB	10.5 MB
MD242	649	8.12 MB	83 KB	162 MB	18.4 MB
MD251	25	23.5 MB	430 KB	136 MB	28 MB
NS40	69	21.7 MB	282 KB	138 MB	27 MB
NS137	110	18.9 MB	870 KB	139 MB	23.3 MB
wxD1-2	42	13.2 MB	356 KB	132 MB	27 MB
wxD1-1	8	23.6 MB	21.7 MB	129.2 MB	29 MB
D. sechellia	378	24.8 MB	131 KB	153 MB	28.8 MB
D. mauritianta	354	24.2 MB	174 KB	154 MB	30.4 MB
