# Supplementary material for: Rapid evolutionary diversification of the flamenco locus across simulans clade Drosophila species
Source: PLoS Genet. 2023 Aug 29;19(8):e1010914. doi: 10.1371/journal.pgen.1010914 (PMC10495008; doi:10.1371/journal.pgen.1010914)
Supplement: S2 Table — (PDF) [file pgen.1010914.s009.pdf]

Species/Strain	Strain	Chr	Start	End	Identity	Status
D. mauritiana	*w12*	X	21407382	22248372	*flamenco*	complete
D. melanogaster	*iso-1*	chrX	21639983	22019657	*flamenco*	complete
D. sechellia	*14021-0248.25*	NW_022611243.1	54590	418077	*flamenco*	complete
D. simulans	*WXD1-1*	X	21641574	22078805	*duplicate*	complete
D. simulans	*WXD1-1*	X	21127235	21547722	*flamenco*	complete
D. simulans	*WXD1-2*	chr_X	21120914	21579070	*flamenco*	complete
D. simulans	*MD251*	chr_X	21031039	21518582	*flamenco*	complete
D. simulans	*MD251*	chr_X	21580275	21986909	*duplicate*	complete
D. simulans	*SZ45*	tig00001037	118032	541006	*duplicate*	complete
D. simulans	*SZ45*	tig00001107	1	341500	*flamenco*	incomplete
D. simulans	*MD242*	tig00000603	500332	929454	*flamenco*	complete
D. simulans	*MD242*	tig00000603	84873	444915	*duplicate*	complete
D. simulans	*NS137*	chr_X_unlocalized	20964026	21241316	*flamenco*	complete
D. simulans	*SZ129*	ig00000300_pilon_pilon	0	397017	*duplicate*	incomplete
D. simulans	*SZ129*	tig00000300_pilon_pilon	464576	895002	*flamenco*	complete
D. simulans	*SZ232*	tig00000724_pilon_pilon	69000	224020	*flamenco*	?
D. simulans	*SZ232*	tig00000792_pilon_pilon	146034	575012	*duplicate*	complete
D. simulans	*LNP-15-062*	tig00000577	116461	644025	*duplicate*	complete
D. simulans	*LNP-15-062*	tig00000577	736039	1206997	*flamenco*	complete
D. simulans	*NS40*	chr_X	20953052	21624701	*flamenco*	complete
D. simulans	*SZ244*	tig00000001_pilon_pilon	0	221382	*flamenco*	incomplete
D. simulans	*SZ244*	tig00000096_pilon_pilon	262120	346650	*duplicate*	incomplete
