# Supplementary material for: Rapid evolutionary diversification of the flamenco locus across simulans clade Drosophila species
Source: PLoS Genet. 2023 Aug 29;19(8):e1010914. doi: 10.1371/journal.pgen.1010914 (PMC10495008; doi:10.1371/journal.pgen.1010914)
Supplement: S3 Table — (PDF) [file pgen.1010914.s010.pdf]

Genotype	Identity	% antisense	% of LTR on antisense
D. mauritiana	*flamenco*	0.71	0.85
D. melanogaster	*flamenco*	0.78	0.82
D. sechellia	*flamenco*	0.78	0.81
wxD1-1	*flamenco*	0.55	0.82
wxD1-2	*flamenco*	0.56	0.77
wxD1-2	*duplicate*	0.57	0.85
LNP-15-062	*flamenco*	0.55	0.86
LNP-15-062	*duplicate*	0.62	0.94
MD242	*flamenco*	0.59	0.85
MD242	*duplicate*	0.72	0.98
MD251	*flamenco*	0.54	0.79
MD251	*duplicate*	0.63	0.78
NS137	*flamenco*	0.51	0.88
NS40	*flamenco*	0.63	0.77
SZ232	*flamenco*	0.51	1.00
SZ232	*duplicate*	0.74	0.96
SZ45	*duplicate*	0.74	0.95
SZ45	*flamenco*	0.60	0.88
SZ129	*flamenco*	0.54	0.84
SZ129	*duplicate*	0.73	1.00

Supplemental Table 3: The percent of annotated TEs that are in the antisense orientation within the *flamenco* region and the % of those antisense TEs that belong to the LTR class of TEs.
